# Consultation-Liaison Psychiatry—from theory to clinical practice: an observational study in a general hospital

**DOI:** 10.1186/s13104-015-1375-6

**Published:** 2015-09-24

**Authors:** Giuseppina De Giorgio, Roberto Quartesan, Tiziana Sciarma, Martina Giulietti, Angela Piazzoli, Laura Scarponi, Silvia Ferrari, Laura Ferranti, Patrizia Moretti, Massimiliano Piselli

**Affiliations:** Functional Homogeneous Area, Local Health Authority 3, Umbria, Italy; Section of Psychiatry, Clinical Psychology and Psychiatric Rehabilitation, Department of Clinical and Experimental Medicine, University of Perugia, Perugia, Italy; Department of Psychiatry, University of Modena and Reggio Emilia, Modena, Italy; Psychiatry School, University of Perugia, Perugia, Italy

**Keywords:** Consultation-Liaison Psychiatry, Comorbidity, Psychological distress

## Abstract

**Background:**

To investigate significant association between various clinical and extra-clinical factors brought out the activities of Consultation-Liaison Service.

**Methods:**

Data from all psychiatric consultations for patients admitted to the Perugia General Hospital and carried out over a 1-year period (from July the 1st 2009 to June the 30th 2010) were collected by a structured clinical report including: socio-demographic features, features of referrals, features of back-referrals. T-test, Mann–Whitney U-test, χ^2^-test and Fischer’s were statistically used.

**Results:**

1098 consultations were performed. The consultations carried out the Emergency Unit were excluded from the study. The type and the reasons for the referrals were discussed such as the ICD-10 diagnosis and the liaison interventions too. Significant associations emerged between gender and: social status and occupation (*p* < 0.05 and *p* < 0.01 respectively). Clinical sector related with reason for referral (*p* < 0.01), type of consultation (*p* < 0.01), liaison investigations (*p* < 0.01) and long-term treatment plan after hospital discharge (*p* < 0.01). The ICD-10 psychiatric diagnosis (Schizophrenia, Affective Syndrome and Neurotic-StressSomatoform Syndrome) was associated with social status (*p* < 0.01), social condition (*p* < 0.01), consultation type (*p* < 0.01), advice (*p* < 0.01) and reason for consultation (*p* < 0.01).

**Conclusions:**

The need for better physical and psychological investigation is confirmed in order to promote not only disease remission but overall wellbeing.

**Electronic supplementary material:**

The online version of this article (doi:10.1186/s13104-015-1375-6) contains supplementary material, which is available to authorized users.

## Background

Over the past two decades research in psychiatry has identified Consultation-Liaison Psychiatry (CLP) as “The guardian of holistic approach to the patient” [1], underlining its pre-eminent role in management of patients who are admitted to a general hospital. The CLP objectives and operating procedures have evolved in recent years from administration of psychiatric treatment [2] to integrating therapy [3] into the bio-psyo-social model along the lines of the recommendation from an editorial in the *Lancet* “No health without mental health” [4]. Hospital staff are confronted daily by physical/psychiatric multimorbidity [5] with its extensive costs of suffering for patient and consumption of medical and economic resources [3]. It is worth noting that psychiatric disorders, even when sub-clinical [6], worsen outcome, lengthen hospital stays and are associated with increased mortality and use of health service resources [7, 8].

Although early detection and treatment of psychological distress and psychiatric disorders in comorbidity are known to reduce health care costs significantly [9], in Italy no shared systematic schema of CLP interventions has, as yet, been drawn up. Whereas CLP was token systematically, various limits appeared, underlying the need to apply new method both inside and outside of general hospital [10, 11].

Moreover, awareness of how interdependent the psychological and physical features of disease are, appears to have widened the gap between research and clinical practice. The former measures outcome efficacy using parameters of little relevance to medicine overall [12], and the latter cannot intervene without taking into account social and health service organization and the network of family and social assistance that obtain [13].

All over the medicine understand that psycho-social features influence aetiopathogenesis and prognosis of many chronic disease such as ischemic heart disease, diabetes, cancer [14]. All over the medicine understand that psycho-social variables are crucial to management of the patient with multi-morbidity and “unexplained medical symptoms”, to the doctor–patient relationship, response to therapy, maintenance of illness behaviour and the onset of psychiatric complications in medical illness [15]. However this awareness has not yet brought significant change in research and particularly in clinical practice, with its two-pronged interventions directed towards patients (in consultation) and towards physicians and surgeons in other hospital units (in liasion). This raises the question of creating multi-disciplinary teams with the psychiatrist mediating CLP integration and system [16]. Furthermore, the unique features of CLP and the difficulties in implementing it are due to patients lacking both awareness of their own psychiatric disturbances and not personally requesting intervention [16].

Regarding research, few reports are available on long-term follow-ups and outcomes. Most studies are retrospective and descriptive [17, 18], and often cannot be compared because of structural and methodological differences [17].

The success of CLP intervention depends on variables such as how the service is organised, the team experience and uniformity of intervention and ability to establish good lines of communication with specialists in internal medicine [19]. The consultant psychiatrist, when called upon to coordinate, inform and educate [16], needs standard procedures, guidelines and quality indicators [20]. This observational study describes CLP activity as conducted in Perugia General Hospital, Italy, with the aims of assessing

- which hospital unit most requested psychiatric consultation;
- what factors were most closely associated with the request (medical and psychiatric diagnosis, drug and psychological therapy) and
- the role bio-psycho-social variables played in determining the above factors.

## Methods

CLP activity was performed by the Psychiatry, Clinical Psychology and Psychiatric Rehabilitation Unit, University of Perugia at Perugia General Hospital, Italy. The hospital has 740 beds for inpatients and Day Hospital/Surgery Services and treats about 43,500 patients per year.

The Psychiatry, Clinical Psychology and Psychiatric Rehabilitation Unit receives requests for urgent or planned consultations, via intranet, from all hospital units (in- and out-patients) and the Accident and Emergency Unit. Urgent requests are prioritized and all consultations are carried out within 24 h of request. Written comments and diagnosis according to Diagnostic and Statistical Manual of Mental Disorders-Text Revision (DSM-IV TR) [21] are provided. For present study purposes DSM-IV TR diagnoses were transformed into ICD-10 diagnoses. Upon patient discharge, the Psychiatry Unit contacts local health services, general practitioners (GPs) and, when required, organises patient transfer to other psychiatric institutes. All these activities are conducted in accordance with ECLW proposals for standardized data collection and training (1996), using a data collection form derived from the Patient Registration Form [22] and full-team participation in weekly supervision sessions [23].

In the present study consultations were carried out between 1st July 2009 and 30th June 2010, 6 days a week from 8.00 a.m. to 8.00 p.m. A pool of psychiatrists from Local Health Board No 2 in Umbria was on call for consultations at other times. All patients were over 18 years of age. To group data, Hospital Units requesting consultation were assigned to one of three sectors: medical, surgical or specialist^1^ (Additional file 1: Table S1).

The data were scheduled into:

- socio-demographic features;
- referrals features (urgency, referrals reason, referring ward, medical co-morbidity);
- referrals result (psychiatric history, psychiatric diagnosis, therapy, pharmacologic prescription).

### Statistical analysis

Continuous variables were analysed by T-test and non-parametric variables by the Mann–Whitney U-Test. Categorical variables were analysed by the χ^2^-tests and Fischer’s exact test. Significance was set at *p* < 0.05. The SPSS programme (version 12.0) was used for data analysis.

## Results

In the mentioned time 1098 psychiatric consultations were carried out (932 first examinations and 166 check-ups).

First examinations: 275/932 (29.5 %) were requested by the Accident and Emergency Unit (A&E) and the other 657 (70.5 %) from other Hospital Units. Excluding A&E requested from the present data analysis because the Unit did not have in-patients, a total of 811 patients were examined in psychiatric consultations. They constituted 1.8 % of the total number of in-patients. 657/811 patients (81 %) received only one examination and 154 (19 %) more than one. Additional file 1: Table S1 reports the socio-demographic characteristics of patients who received psychiatric assessment through consultation.

Almost all the inpatients were from Europe, the mean age was 57.9 (SD ± 19.4); the female were 60.8 %; married people were 47.5 %; in most cases (39.5 %) the inpatients were pensioner.

Details of consultations: 24.7 % of requests were flagged as urgent and 84.6 % of patients had been informed of the request. Medical units provided 53.1 % of requests for psychiatric consultation, surgical units 8.8 % and specialist units 38.1 %. The most common reasons for requesting consultation were anxiety (18.9 %), symptoms of depression (18.2 %), confusion (13.4 %), unexplained somatic symptoms (11.2 %), suicide attempt/risk (11.2 %), psychomotor agitation (10.9 %) and history of psychiatric illness (14.4 %). Reasons from medical units focused on anxiety (22.3 %) and depression (16.9 %) (Additional file 1: Table S2).

The main reasons for consultation from surgical units were suicide attempt/risk (31 %) and agitation (21.1 %) (Additional file 1: Table S2). The specialist units motivated requests on the grounds of depression (21.7 %), unexplained somatic symptoms (17.8 %) and anxiety (159 %) (Additional file 1: Table S2).

Referrals result: A history of psychiatric illness was present in 41.3 % of patients and 60.8 % used psychoactive drugs (usually benzodiazepine and/or antidepressant agents). Only 17.5 % of patients was being treated by the health service when hospitalised. The most common ICD-10 diagnoses (International Classification of Disease) were Somatic–Neurotic–Stress Syndrome (28.7 %) and Affective Syndromes (26.6 %).

The psychiatric consultation was the only diagnostic intervention in 83.3 % of cases. Liason interventions were mainly directed towards medical and nursing staff of the Unit requesting consultation (66.2 %). Therapeutic interventions included patient interviews (47.5 %) and prescription of psychoactive drugs (60.3 %). Benzodiazepine and antidepressant were the most prescribed drugs. However, use of BDZ as monotherapy was reduced from 14.5 % at admission to 8.1 % and monotherapy with neuroleptic agents was increased from 6.2 % at admission to 8.2 %. The discharge plan of care included referral to the CSM (24.8 %), to the Psychiatric Unit day service (20.1 %) or to the patient’s GP (17.1 %).

### Associations between variables

Significant associations emerged between gender, the two options for marital status (*p* < 0.05), occupational status (*p* < 0.01) and information about the consultation (*p* < 0.05). Males were more frequently single and in work than females (Additional file 1: Table S3).

Sources of requests for consultation correlated significantly reasons for requests (*p* < 0.01), the type of consultation (*p* < 0.01), interventions for diagnosis (*p* < 0.01), liason (*p* < 0.01) and discharge programme (<0.01).

Patients who had attempted or were at risk of suicide were more often admitted to surgical units which most frequently requested urgent consultations (Additional file 1: Table S4).

The main ICD-10 psychiatric diagnoses (schizophrenia, affective or neurotic-stress-somatoform syndromes) were significantly associated with marital status (*p* < 0.01), socio-environmental status (*p* < 0.01), type of consultation (*p* < 0.01), patient information (*p* < 0.01) and reason for request (*p* < 0.01).

Post hoc analysis showed that patients with schizophrenia lived with their original families, were single, pensioners or invalids, assessed in urgent consultations, less informed than others and directed to the CSM when discharged, after having been put in contact with the CSM during the consultation (Additional file 1: Table S5).

For patients with affective syndromes, suicide attempt or risk and depression were the reasons for requesting consultation. They were directed to their GP upon discharge from hospital (Additional file 1: Table S5). Patients with neurotic-stress-somatoform syndromes had jobs and were assessed because of anxiety and unexplained medical symptoms. The main liason intervention was raising awareness among medical and nursing staff in the unit where they were being treated. Upon discharge they were directed to their GP or to the Psychiatry Unit Out-patient Service (Additional file 1: Table S5).

## Discussion

The present study used a CLP service that was structured according to setting and codified assessment procedures [24]. As features satisfied some recommendations that have been reported in other papers the service may thus be considered valid for implementing compliance with CPL interventions and evaluating their effectiveness [19].

In particular referring to description of population and clinical variables the authors close:

- sending requests by intranet ensured the request is clearly stated, and facilitated identifying the most appropriate form of intervention and measurement tools [16];
- performing consultation within 24 h of request improved treatment compliance [19];
- adopting the “clinimetric” [25] rather than the “psychometric” approach led to detection of sub-clinical symptoms and of deficits in some functional areas that persisted after treatment [26];
- applying qualitative rather than quantitative parameters provided a more accurate definition of outcomes [27];
- writing down consultation findings encouraged communication within the multidisciplinary team [16];
- finally, as one feature of the CLP service was a link with the community health and social services, information could be provided about treatment plans after discharge from hospital [14].

Details of consultations: Consultations were requested for 1.8 % of all hospital admissions, which was in line with reports over the years of 1–2 % requests [28]. As observed elsewhere, most patients received only one consultation examination and required psychiatric treatment while in hospital [29].

Social backgrounds and demographic data from patients in the present study were also in line with other reports [30].

Adequate information was imparted to 80 % of patients during the consultation, which is a rise in the 66 % reported in an earlier Italian study by Gala [31]. The improvement may be due to World Health Organization (WHO) campaign which emphasizes that Mental Health is a crucial part of psycho-physical well-being [32]. The present study showed that schizophrenia was the only factor impacting upon lack of patient information, unlike a previous study of ours which had correlated male gender with poor patient information [33]. Provision of information to the patient with schizophrenia may be hampered not only by the patient’s psychopatology such as limited insight and sometimes bizarre behaviour studio [34], but also by the urgent status of consultation requests because of the patient’s agitation or distorted perception of, and contact with, reality [35]. The stigma arisen from referring area is to be confirmed.

### Associations between variables

The high prevalence of patients with affective and neurotic-stress somatoform syndromes in medical and specialist units focuses attention on physical/psychiatric multi-morbidity and the difficulties in medical and psychiatric managing patients with somatisation or with somatic expression of psychological pain [36]. In the surgical sector only about 20 % of requests were motivated by agitation due to physical distress [37], which illustrates the need for flexible, personalised interventions that not only satisfy the demands of patients and medical staff but which can also be adjusted to fit diverse clinical situations [38].

Findings in the present study show the usage rate of an in-depth diagnostic flow chart with psychometric and laboratory tests overlaps with Italian [31] and European [30] reports. Although more drugs were prescribed in interventions than is the trend in Europe, the drug prescription rate was still below what Gala reported in 1999. In any case drug treatment was the most common form of intervention [39], followed by staff support. In general, the most frequently prescribed drugs were anti-depressive agents and BDZ [31].

It is hard to compare the 38 % rate of expressive/supportive interventions which emerged in the present study with other reports because parameters vary greatly [40]. Even though reports demonstrate that in the hospital setting this form of intervention is more effective than interventions directed towards personal or family support or focal psycho- education or therapy, few data are available on the prevalence of expressive/supportive interventions probably because the use of such techniques in CLP has not been standardized. Hunter et al. [40] suggested developing a multi-focal setting to identify pre-morbidity and stressor factors that interfere with patient acceptance of illness and hospitalization and which are the focus of therapy.

Finally, a much lower rate of patient transfer to psychiatric units than reported elsewhere [41], and promotion of treatment plans after discharge from hospital provide evidence of the success the present approach, which aimed at reducing staff anxiety and promoting total care of the patient.

## Conclusions

Hospitalization in itself is a stressful event which may disturb balance of mind in some cases and worsen the clinical condition of patients who are suffering from psychiatric co-morbities [42]. Without specialized training medical and nursing staff may not recognize psychological distress and consequently delay early intervention [43]. A CLP service is able to raise awareness among health service personnel and improve detection of psychological problems in patients, both of which have beneficial repercussions on the length of hospital stays and the well-being of patients. CLP, however, still needs to develop clinical and research standards that are in line with the trend towards Evidence Based Medicine. Innovative methodological tools are required to establish qualitative parameters [44].

A systemic schema of interventions will censure psychiatrists confront the full complexity of diagnosis and therapy, promote all round care of the individual patient and use liason interventions to foster multidisciplinary teamwork.

## Additional file

**Additional file 1.** Data analysis of clinical investigation.

## Footnotes

1 MEDICAL SECTOR: internal medicine and endocrine science; occupational medicine; internal medicine, angiology and atherosclerosis; internal medicine and oncologic ward; internal and vascular medicine ward. SURGICAL SECTOR: intensive care; heart surgery; general surgery; thoracic surgery; vascular surgery; orthopedics; neurosurgery. SPECIALISTIC SECTOR: cardiology; dermatology; hematology; gastroenterology; geriatric ward; infectious medicine; nephrology; ophthalmology; oncologic ward; otorhinolaryngology; obstetrics and gynecology; neurology; lung ward; urology; spinal unit.
