# Supplementary material for: Consultation-Liaison Psychiatry—from theory to clinical practice: an observational study in a general hospital
Source: BMC Res Notes. 2015 Sep 24;8:475. doi: 10.1186/s13104-015-1375-6 (PMC4582719; doi:10.1186/s13104-015-1375-6)
Supplement: Supplementary file 1 — Additional file 1. Data analysis of clinical investigation. [file 13104_2015_1375_MOESM1_ESM.pdf]

TABLE 1 SOCIO-DEMOGRAPHIC FEATURES

	MEAN	SD
**Age**	57.6	±19.4
**Studying years**	9.7	±4.6
	**N**	**%**
**Sex**		
M	318	39.2
F	493	60.8
**Marital status**		
matched	385	47.5
Single	167	20.6
Widower	141	17.4
Separated	69	8.5
Unknown	49	6.0
**Environmental condition**		
Married family	415	51.2
Parental family	98	12.1
Alone	164	20.2
Other condition	60	7.4
In hospice	14	1.7
Unknown	60	7.4
**Employment**		
Pensioner	320	39.5
Employed	211	26.0
Unemployed	70	8.6
Housewife	72	8.9
Student	22	2.7
Unknown	60	7.4
**Origin**		
Europe	748	92.2
Out of Europe	63	7.8

TABLE 2 DETAILS OF CONSULTATION

	MEDICAL SECTOR (M)		SURGICAL SECTOR (SU)		SPECIALISTIC SECTOR (SP)	
	n	%	n	%	n	%
REFERRAL TYPE						
Planned	340	78.9	37	52.1	234	75.7
Urgent	91	21.1	34	47.9	75	24.3
Total	431	100.0	71	100.0	309	100.0
REFERRAL REASON						
Suicide attempt/risk	52	12.1	22	31.0	17	5.5
Anxiety	96	22.3	8	11.3	49	15.9
Depression	73	16.9	8	11.3	67	21.7
Agitation/delirium	59	13.7	15	21.1	35	11.3
Delusion/hallucination	17	3.9	1	1.4	15	4.9
Aggressive behaviour	6	1.4	3	4.2	9	2.9
At the request of patient	2	.5	0	0	5	1.6
Unexplained medical symptom	36	8.4	0	0	55	17.8
Managment and compliance difficult	6	1.4	0	0	11	3.6
Psychiatric hystory	67	15.5	11	15.5	39	12.6
Drugs abuse	17	3.9	3	4.2	7	2.3
Total	431	100.0	71	100.0	309	100.0
ICD-10 DIAGNOSIS						
None	54	12.5	9	12.7	54	17.5
Early onset syndrome	4	.9	0	0	3	1.0
Psycho-organic syndrome	36	8.4	12	16.9	26	8.4
Psichological and behavioural factors associated with dosorder or disease classified elsewere	9	2.1	3	4.2	14	4.5
Substance dependance and abuse	25	5.8	4	5.6	16	5.2
Schizophrenia	31	7.2	3	4.2	10	3.2
Affective Disorders	119	27.6	21	29.6	76	24.6
Neurotic-Stress related-Somatoform disorders	117	27.1	15	21.1	101	32.7
Personality disorders	36	8.4	4	5.6	9	2.9
Total	431	100.0	71	100.0	309	100.0

TABLE 3 : X^2-TEST VARIABLES SIGNIFICANT ASSOCIATE WITH THE GENDER

	**M (%)**	**F (%)**	**p-value**
**Marital status**			
Single	25.5	17.4	<0.05
Widower	12.9	20.3	<0.05
**Employment**			
Employed	32.1	22.1	<0.01

TABLE 4 FISCHER'S TEST AND POST-HOC ANALYSIS. SOCIO-DEMOGRAFIC AND CLINICAL VARIABLES (REFERRAL FEATURES AND REFERRAL RESULTS) SIGNIFICANT ASSOCIATE WITH REFERRING WARD

	**MEDICAL SECTOR (M)**	**SURGICAL SECTOR (SU)**	**SPECIALISTIC SECTOR (SP)**	**p-value**	**post-hoc**
**Referrals reason**				<0.01	
Suicide risk/attempt	12.0	31.0	5.5		SU>M,SP
Anxiety	22.3	11.3	15.9		M>SU,SP
Depression	16.9	11.3	21.7		SP>M,SU
Agitation/delirium	13.7	21.1	11.3		SU>M,SP
Unexplained medical symptom	8.4	0.0	17.8		SP>SU,M M>SU
Management and compliance difficulties	1.4	0.0	3.6		SP>M,SU M>SU
**Referrals type**				<0.01	
Urgent	21.1	47.9	24.3		SU>M,SP
**ICD-10 diagnosis**				<0.05	
Psycho-organic syndrome	8.4	16.9	8.4		SU>M,SP
Schizophrenia	7.2	4.2	3.2		M>SU,SP
Neurotic-Stress Related-Somatoform Disorders	27.1	21.1	32.7		SP>M,SU
**Intervention for diagnosis**				<0.01	
Laboratory test and imaging (EEG, CT, MNR)	57.1	4.1	26.5		M> SU,SP SP>SU
**Intervention for liaison**				<0.01	
local public psychiatric service (Mental Health Service –italian acronym CSM-)	4.9	12.7	2.3		SU>M,SP
general practitioner	1.0	0.0	0.0		M>SU,SP
Referring staff	64.3	57.7	69.9		M,SP>SU
**Plans after discarge**				<0.01	
Sending to general practitioner	20.0	5.6	14.9		M> SU,SP SP>SU

TABLE 5 FISCHER'S TEST AND POST-HOC ANALYSIS SOCIO-DEMOGRAFIC AND CLINICAL VARIABLES (REFERRAL FEATURES AND REFERRAL RESULTS) SIGNIFICANT ASSOCIATE WITH ICD-10 DIAGNOSIS

	**Schizophrenia (S)**	**Affective Syndrome (AS)**	**Neurotic-Stress Related Somatoform Syndrome (NS)**	**p**	**post-hoc**
**Marital status**				<0.01	
Single	54.8	18.6	30.6		S>AS,NS
**Environmental condition**				<0.01	
Parental family	29.5	6.9	19.8		S>AS,NS
**Employment**				<0.05	
Employed	11.4	26.4	32.6		NS>S,AS<br>AS>S
Pensioner (includin disability)	52.3	40.3	36.5		S>AS,NS
**Type of referral**				<0.01	
Urgent	38.6	24.5	16.7		S>AS,NS
**Advice to the patient regarding the consultation**				<0.01	
No	31.8	12.0	7.3		S>AS,SN
**Referrals reason**				<0.01	
Suicide risk/attempt	4.5	16.7	8.2		SA>S,SN
Anxyeti	6.8	12.5	31.8		SN>S,SA
Depression	4.5	31.9	15.5		SA>S,SN<br>SN>S
Delirium	20.5	6.9	10.7		S>SA,SN
Delusion/hallucination	34.1	1.9	1.3		S>SA,SN
Unexplained medical symptoms	4.5	5.6	16.7		SN>S,SA
**Intervention of Liaison**				<0.05	
local public psychiatric service (Mental Health Service-italian acronym CSM)	15.9	7.4	1.7		S>SA,NS
Referring staff	50.0	64.4	74.7		NS>S,AS<br>AS>S
**Plan after discarge**				<0.05	
Sending to CSM	63.6	31.0	20.2		S>AS,NS<br>AS>NS
General practitioner	2.3	16.7	22.7		AS>S<br>SN>S
Outpatient clinic (Perugia University)	6.8	28.2	30.5		AS>S<br>NS>S
